# Associations of the Mediterranean diet during pregnancy with impaired glucose tolerance and gestational diabetes: A national prospective cohort study in Lebanon

**DOI:** 10.1038/s41430-026-01708-3

**Published:** 2026-03-03

**Authors:** P. Hage Boutros, M. Bassil, J. El Hayek Fares, K. G. Koski

**Affiliations:** 1 Department of Nutrition, Faculty of Health Sciences, Modern University for Business and Science, Beirut, Ramiz Sarkis, Beirut, 1137501 Lebanon; 2 Department of Nutrition Sciences, College of Health Sciences, QU Health, Qatar University, Doha, Qatar; 3 Faculty of Nursing and Health Sciences, Notre Dame University- Louaize (NDU), Zouk Mosbeh, Lebanon, PO Box:72, Zouk Mikael, Lebanon; 4 School of Human Nutrition McGill University - Macdonald Campus 21111 Lakeshore Road, Ste Anne de Bellevue, Quebec, H9X 3V9 Canada

**Keywords:** Gestational diabetes, Risk factors

## Abstract

**Background/objectives:**

This study assessed whether adherence to the Lebanese Mediterranean diet (LMeD) is associated with a lower risk of gestational diabetes mellitus (GDM) and impaired glucose tolerance (IGT) among a national sample of pregnant women in Lebanon.

**Methods:**

For this longitudinal study, 618 women were recruited in trimester 1. The main outcomes were IGT and GDM. Independent variables included adherence to the LMeD, maternal anthropometry, clinical and biochemical variables collected at 3 trimesters. Hierarchical multiple logistic regression was used to test associations of independent predictors with IGT and GDM.

**Results:**

The diagnosis of GDM was 5.6%, whereas IGT was more prevalent, and increased from 17% to 24% from T1 to T3, but neither GDM nor IGT risk was associated with adherence to LMeD. A higher consumption of legumes and burghol increased IGT risk in trimester 1 whereas vegetables lowered IGT risk in trimester 3. Family history of diabetes, high gestational weight gain (GWG) and elevated Mean Arterial Pressure (eMAP) were associated with increased GDM risk.

**Conclusion:**

Findings underscore the importance of early screening for family history of diabetes, excessive gestational weight gain (GWG), stress, and elevated mean arterial pressure (MAP) to target to identify women at risk of IGT and GDM. Trimester-specific dietary strategies, such as reducing overconsumption of burghul and legumes in early pregnancy and promoting vegetable intake later, may help improve maternal glycemia.

## Introduction

Dietary patterns, particularly adherence to the Mediterranean diet (MeD) which is characterized by high consumption of vegetables, fruits, nuts and extra virgin olive oil are increasing in popularity due to numerous health benefits during pregnancy [1]. More than 22 indexes have been developed specific for Mediterranean countries to assess adherence to the MeD in the general population and among these, several were modified for use with pregnant women by removing alcohol from the scoring and/or including micronutrients needed for pregnancy such as calcium, folic acid, and iron [2]. The MeD has consistently been linked with healthier gestational weight gain (GWG), reduced cardiometabolic risk factors [2], and lower risk of gestational diabetes mellitus (GDM) [1].

Growing evidence suggests that the MeD can reduce both the risk of GDM and impaired glucose tolerance [1]. Three large clinical trials reported a significant reduction in GDM risk by using a MeD intervention in early pregnancy [3], a MeD diet supplemented with extra virgin olive oil (EVOO) and pistachios [4] and MeD adherence to the following 6 food items: >12 servings/week of vegetable and fruits, 3 servings/week of nuts, >6 days/week consumption of extra virgin olive oil (EVOO), and ≥40 mL/day of EVOO [5]. More recently, a study reported a 41% reduction in GDM risk among pregnant women who ranked in the highest tertile for adherence to the MeD early during pregnancy [6]. Moreover, a large prospective cohort study covering 10 Mediterranean countries reported a decrease in GDM risk among women with high adherence to the MeD prior to the OGTT test at 24–32 weeks, as well as improved glucose tolerance in women without GDM and better adherence to the MeD [7].

Psychosocial factors, such as stress, depression, and poor sleep, are increasingly recognized as important determinants of GDM and impaired glucose tolerance [8]. Depression and high stress levels are particularly prevalent in the Middle Eastern populations and may exacerbate insulin resistance and GDM risk [9]. Additionally, psychosocial stress often leads to poor dietary choices, reduced physical activity, and higher weight gain during pregnancy further contributing to GDM [10].

Despite greater likelihood of adherence to the MeD, Middle Eastern and North African (MENA) countries report some of the highest rates of gestational hyperglycemia worldwide (22.3%), partly due to rapid dietary westernization [11]. In Lebanon, adult diabetes and impaired glucose metabolism are among the highest globally [12]. Yet, this might not translate to pregnancy-specific hyperglycemia or GDM, as these conditions remain understudied and their determinants poorly characterized. In addition, trimester-specific predictors need to be further ivestigated in this region, as maternal weight gain, diet, and psychosocial factors vary throughout pregnancy and may influence GDM risk differently at each stage. The traditional Lebanese Mediterranean Diet (LMeD) shares core MeD features but includes region-specific foods such as burghul, legumes, and vegetables, which may influence carbohydrate quality and glycemic load [13]. One study showed a reduction of diabetes risk with higher LMed adherence in the general population [14], yet this association remains underexplored among pregnant women. Understanding these dietary specifics is particularly important given their potential impact on gestational weight gain and impaired glucose tolerance. Therefore, evaluating LMeD adherence alongside broader dietary dimensions, including macronutrient distribution and refined versus complex carbohydrates, would provide a better understanding of diet quality in relation to glucose metabolism during pregnancy. Taken together, it is important to understand the interplay of maternal factors including pre-pregnancy weight, GWG, LMeD diet and psychosocial factors in the three different gestational trimesters in managing the risk of GDM and impaired glucose tolerance (IGT) within the Lebanese context. Therefore, this national prospective cohort study aimed to (1) estimate the prevalence of GDM and IGT among pregnant Lebanese women, (2) identify trimester-specific predictors, and (3) evaluate the impact of LMeD adherence. We hypothesized that poor LMeD adherence, excessive GWG, and adverse psychosocial factors would increase the risk of GDM and IGT.

## Materials and methods

### Study population

This national prospective longitudinal study recruited pregnant women living in the 6 different governorates of Lebanon: Mount Lebanon, Beirut (capital of Lebanon), Bekaa, South (and Nabatieh), North, and Akkar. A simple random sampling among all obstetric clinics in Lebanon was done using the statistical software SPSS (Statistical Package for Social Sciences), version 22.0. Of the 732 obstetric clinics, a total of 20 private and hospital based private clinics were selected using simple random sampling: Mount Lebanon (*n* = 3), Beirut (*n* = 5), Bekaa (*n* = 4), South (*n* = 3), North (*n* = 4), and Akkar (*n* = 1).

A systematic approach was used to recruit approximately 30 women per clinic. Clinic staff generated a list of all eligible patients attending prenatal visits during the recruitment period, and participants were selected using a simple random sampling approach. Women were approached in person, provided with detailed study information, and asked to provide written informed consent. Recruitment was conducted between 2019 and 2021 and coincided with the COVID-19 outbreak and Lebanese economic crisis. Consenting pregnant women were followed from the 1st trimester (<15 weeks of gestation) until delivery. The initial recruitment interview was conducted in person with follow-up interviews by phone in the second (24–28 weeks), and third trimesters (34–37 weeks).

We used Epi Info Software to calculate the sample size needed using the formula by Fleiss with correction [15]. The confidence interval was set at 95%, power at 80% and ratio (unexposed/exposed to the MeD=1). A sample of 618 participants was targeted after adjusting for a 20% loss to follow up. Of the 660 participants, 42 dropped out due to miscarriages and/or other reasons.

Inclusion criteria were pregnant Lebanese women > 18 years with a singleton pregnancy. Exclusion criteria included women with multiple gestations, or pre-pregnancy diabetes, or those carrying a fetus with structural malformation, chromosomal anomalies or TORCH (toxoplasmosis, rubella, cytomegalovirus, herpes and other agents) infections.

### Study design

A survey was administered which included a section on socio-demographic data. Validated questionnaires were used to assess stress [16], sleep [17], depression [18], adherence to the LMeD [13] and physical activity [19] at T1, T2 and T3. Clinical data was obtained through medical charts, including self-reported pre-pregnancy weight, vitamin/mineral intake, presence of anemia and medical history (maternal previous GDM and family history of diabetes). At follow-up visits at each trimester, additional clinical indicators were collected from medical charts including IGT per trimester, diagnosis of GDM, and general maternal health status (anemia, vitamin/mineral supplementation, COVID). The main exposure variable was LMeD adherence which was assessed in each trimester. The main outcomes were: (1) diagnosis of GDM between 24 and 28 weeks and (2) IGT in T1 and T3. IRB approval was obtained from Bellevue Medical Center’s institutional review board in accordance with the 1964 Helinski Declaration [20]. Written consent was obtained from each physician at the 20 private clinics in order to recruit their patients and access medical records.

### Questionnaires

#### Dietary assessment and adherence to the Mediterranean diet

Food intake was collected using a validated 61-item food frequency questionnaire (FFQ) for the Lebanese diet as it incorporates traditional Lebanese dishes [13]. This FFQ consists of 9 food categories including (1) breads and cereals, (2) dairy products, (3) fruits and juices, (4) vegetables, (5) meat and alternates, (6) fats and oils, (7) sweets and desserts, (8) beverages, and (9) miscellaneous.

This LMeD pattern consisted of 9 food groups that had high loading on this pattern which included: fruits, vegetables, legumes, olive oil, burghol (crushed whole wheat), milk and dairy products, starchy vegetables (potato, corn and beans), dried fruits and eggs [13]. Adherence to the LMeD was measured based on the consumption of each of the nine food groups with 1, 2 and 3 points assigned to intakes in the 1^st^, 2^nd^ and 3^rd^ tertile indicating low, medium and high intake, respectively. Scores ranged between 9 and 27, with 9 reflecting the least adherence and 27 reflecting the highest adherence to the LMeD [13]. Alcohol intake was excluded as consumption is virtually absent in this study population. This tool showed a good correlation (*r* = 0.56, *p* < 0.01) with the Italian MeD adherence tool [21].

#### Edinburgh perinatal/postnatal depression scale (EPDS)

Edinburgh Perinatal/Postnatal Depression Scale (EPDS) is a commonly used 10-item scale in clinical practice to identify women at risk of perinatal depression. A global sum of 0-30 is obtained. A score of 10 or greater indicates possible depression [18]. It has been validated in a Lebanese pregnant population with Cronbach’s alpha=0.84, correlation (*r* = 0.57, *p* < 0.01) with the Present State Examination [22].

#### Perceived stress scale (PSS10)

Perceived stress was evaluated using the Perceived Stress Scale (PSS10), which was later shortened to 10 items [16]. It has been validated in a Lebanese pregnant population with test-retest reliability and Cronbach’s alpha=0.74; Correlation r = 0.48, *p* < 0.001 and *r* = 0.58, *p* < 0.001 with a general health questionnaire and EPDS, respectively [14]. A global sum score of 0–40 is obtained with higher scores indicating higher perceived stress. The categories are: Low stress (score 0–13), moderate stress (score 14–26), and high stress (score 27–40).

#### Pittsburgh Sleep Quality Index (PSQI)

PSQI is a self-reported tool that assesses sleep quality over a 1-month period. A global score ranges between 0 and 21. A score of equal or greater to 5 indicates poor sleep, while a score of less than 5 indicates good sleep. It has been validated in an Arabic population with Cronbach’s alpha=0.74, correlation *r* = 0.36, *p* < 0.001 and *r* = 0.19, *p* = 0.03, with the Insomnia Severity Index and Medical Outcome Study Short Form, respectively [17].

#### Canadian Physical Activity Readiness Examination (PARmed-X)

PARmed-X was used to assess physical activity in the past month [19]. Participants were categorized according to the frequency, intensity and duration of physical activity. Accordingly, categories were “sedentary” (frequency <1-2 times/ week and <20 min duration; PA index=0), and “active” (1-2 times/ week for 20 min or more than twice/week for < 20 min; PA index=1 or >2 times/week for more than 20 min; PA index=2).

### Clinical measurements

#### Pre-pregnancy Body Mass Index (BMI) and Gestational Weight Gain (GWG)

Pre-pregnancy BMI was collected at baseline from obstetrical charts and was used to classify women as underweight (<18.5 kg/m^2^), normal (18.5–24.9 kg/m^2^), overweight (25-29.9 kg/m^2^) or obese (≥30 kg/m^2^). Total GWG in Kg was calculated by subtracting pre-pregnancy weight from weight at 39 weeks gestation, and GWG was classified as low, adequate or excessive according to the pre-pregnancy BMI using the Institute of Medicine (IOM) recommendations (underweight 12.5-18 kg, normal weight 11.5-16 kg, overweight 7-11.5 kg, and obese 5-9 kg). GWG per trimester was also calculated and classified as adequate, excessive or low according to the following recommendations: For 1st trimester: above 2 kgs or below 1 kg was considered excessive or low, respectively for all categories of BMI. For 2nd and 3rd trimester, the recommended weight gain per week for each BMI category is the following: 0.45–0.6 kg/wk for underweight women, 0.35–0.45 kg/wk for normal weight women, 0.2–0.3 kg/wk for both overweight, and obese women. Weight gain below or above these values was classified as low or excessive, respectively [23].

#### FBG, IGT and GDM

FBG values were collected from obstetric charts at T1 and T3, and were categorized into normal FBG: < 100 mg/dl and IGT ≥ 100 mg/dl, while the presence of GDM diagnosis (yes/no) was confirmed during the visit in the second trimester [24]. Obstetricians in Lebanon followed the two-step 50 g oral glucose tolerance test between 24-28 weeks in line with the American College of Obstetricians and Gynecologists [25]. If blood glucose was ≥140 mg/dL one hour after the intake of 50 g glucose, a 100 g three-hour oral glucose tolerance test was performed. In the latter, blood glucose was measured at t = 0 (fasting), t = 1 h, t = 2 h and t = 3 h with cut-off values (Carpenter and Coustan criteria) of 95, 180, 155, and 140 mg/dL, respectively [23]. GDM diagnosis was made when two or more blood glucose levels met or exceeded the cut-off values [24].

#### Mean Arterial Pressure (MAP)

MAP is a novel blood pressure index that was more sensitive in predicting abnormal blood pressure in this population. It is computed using systolic and diastolic blood pressures (SBP and DBP, respectively) and can be elevated despite mothers having SBP AND DBP within normal ranges. MAP is calculated using the formula. MAP = DBP + 0.33 [SBP-DBP] (25). Trimester-specific cutoffs for elevated MAP (eMAP) in pregnancy are defined as >87 mmHg (10- < 18 weeks), >84 mmHg (18-34 weeks), and >86 mmHg (after 34 weeks), while low MAP is defined as <70 mmHg [26].

### Statistical analyses

Univariate analyses (measures of central tendency, percentage and frequencies) were used to summarize the population characteristics of pregnant women as well as the frequency of consumption of each food group of the LMeD. Diet, GWG, smoking, physical activity, and psychosocial factors including stress, sleep and depression were compared across the trimesters using the McNemar test. Frequencies and percentages were used to determine the categorical dietary variable: adherence to the LMeD, while means were used to determine mean adherence of the population as well as mean intake of each of the 9 food groups of the LMeD. Scatter plots were used to describe the FBG measurements in trimester 1 and 3. Maternal characteristics were also compared among women with normal FBG (<100 mg/dl) vs. IGT (≥100 mg/dL) in trimesters 1 and 3.

Hierarchical logistic regression models were used to test the associations between predictors and elevated FBG in trimesters 1 and 3, while in trimester 2, models were used to test the associations between GDM and the same predictor variables. In model 1, GWG, pre-pregnancy BMI, family history of diabetes, stress, sleep, depression and MAP were entered. In model 2, adherence to the LMeD was added, and in model 3, each of the 9 food groups in individual models along with the significant variables from model 2 were entered. All the analyses were conducted using SAS V9.4.

## Results

### General population characteristics

Table 1 details the characteristics of the 618 pregnant women. Nearly half (43%) resided in urban centers (Mount Lebanon and Beirut); the remainder lived in rural communities. Participants’ average age was 29.2 ± .5.0 yrs. Most (63.8%) had a normal pre-pregnancy BMI but 32.4% were overweight or obese. The majority were sedentary with prevalence of 84%, 89%, and 95% in the first, second and third trimesters, respectively. Additionally, 68.3% of the participants gained weight in excess of IOM recommendations. Most did not have a history of GDM (98%) or hypertensive disorders (96.4%); the incidence of GDM in the current pregnancy was low (5.6%). Among women diagnosed with GDM at T2, 50% already had IGT in the first trimester.

**Table 1:** Population characteristics of a national sample of pregnant women in Lebanon.

Variable	Mean ± SD or %
**Maternal characteristics**	
^*^ Age, years	29.2 ± 5.0
Parity	1.3 ±1.1
^†^ Place of Residence	
Akkar	3.7
Beirut, %	12.4
Bekaa, %	16.4
Mount Lebanon, %	30.7
North Lebanon, %	7.4
South Lebanon, %	29.4
Occupation	
Student, %	1.1
Homemaker, %	49.2
Self-employed, %	8.7
Employee part-time, %	9.8
Employee full-time, %	31.2
Education	
High school or less, %	23.9
Bachelor degree, %	40.6
Masters degree or higher, %	35.4
**Maternal Health Status**	
^‡^ Pre-pregnancy BMI, kg/m^2^	
Underweight (<18.5, %)	3.9
Normal (18.5-24.9, %)	63.4
Overweight (25.0-29.9, %)	22.5
Obese (≥30.0, %)	10.2
^§^ Total GWG, kg	
Low, %	11.1
Adequate, %	20.5
Excessive, %	68.3
Previous Hypertensive Disorders of Pregnancy, % Yes	3.6
Family History of Diabetes, % Yes	20.8
Previous GDM, % Yes	1.9
^||^ Current GDM, % Yes	5.6
^¶^ IGT 1^st^ trimester, % Yes	12.4
^¶^ IGT 3^rd^ trimester, % Yes	26.5
Vitamin Supplement Intake, % Yes	98.8
OTC Multivitamins/minerals	68.9
Iron	30.8
Calcium/Vitamin D	34.4
^#^ Current Anemia, % Yes	25
COVID Infection during Pregnancy, % Yes	7.7

*Sample size = 618. Values are means ± standard deviation (SD) if normally distributed, median (min-max) if not normally distributed, or percentages (%) if binary, unless otherwise specified.

^†^South Lebanon includes the sample of Nabatieh. ^‡^ Pre-pregnancy BMI was calculated as weight in Kg over height in meters squared, and was classified as underweight, normal, overweight and obese. ^§^Total gestational weight gain was calculated by subtracting total weight gained from pre-pregnancy BMI. GWG was classified as low, adequate or excessive applying the following recommendations: To be considered adequate GWG, women who are underweight should gain a total of 12.5–18 kg, normal weight between 11.5 and 16 kg, overweight between 7-11.5 kg, and obese between 5-9 kg. Those with low GWG have a weight gained below these recommendations whereas those with excessive weight gain have above these recommendations (American College of Obstetricians and Gynecologists, 2013). ^||^ GDM diagnosis was made using the two-step 50 g oral glucose tolerance test between 24-28 weeks in line with the American College of Obstetricians and Gynecologists for diagnosis of GDM. If the 50 g glucose tolerance test was ≥140 mg/dL after one hour, a 100 g 3-h oral glucose tolerance test was done. Blood glucose was tested at t = 0 (fasting), t = 1 h, t = 2 h and t = 3 h with cut-off values of 95, 180, 155, and 140 mg/dL, respectively. GDM diagnosis was made when two or more these cut-off values met or exceeded the Carpenter and Coustan criteria. ^¶^ Impaired glucose tolerance (IGT) was diagnosed with FBG ≥ 100 mg/dl in trimester 1 and 3 (Carpenter and Coustan, 1982). ^#^ Serum ferritin concentration <30 μg/L together with an Hb concentration <11 g/dL was used to determine the presence of anemia during the 1st trimester of pregnancy (Api et al. 2015).

*BMI* body mass index, *GWG* gestational weight gain, *GDM* gestational diabetes mellitus, *IGT* impaired glucose tolerance, *OTC* over the counter medication.

**Fig. 1:**
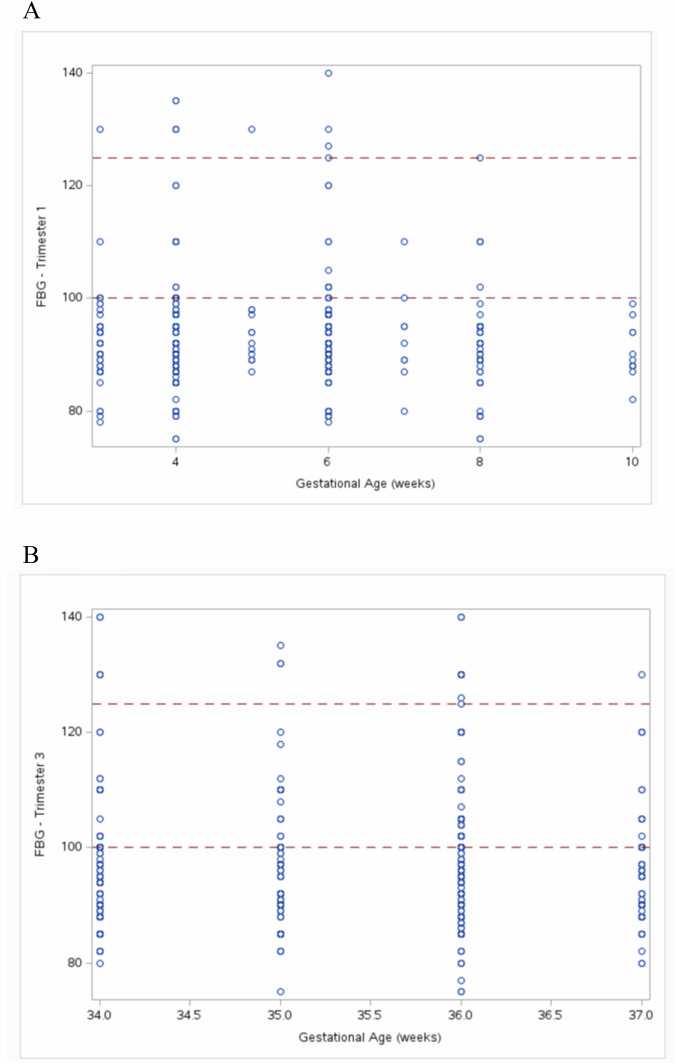
Fasting Blood Glucose Measurements in the First and Third Trimesters of Pregnancy among a National Sample of Pregnant Women in Lebanon. **A** Scatter plot of fasting blood glucose in pregnant women (n = 543) in trimester 1. Dashed lines represent the cut-offs for FBG. Normal <100 mg/dL, impaired glucose tolerance: 100-125 mg/dL, and diabetes ≥126 mg/dL. **B** Scatter plot of fasting blood glucose in pregnant women (n = 559) in trimester 3. Dashed lines represent the cut-offs for FBG. Normal <100 mg/dL, impaired glucose tolerance: 100-125 mg/dL, and diabetes ≥126 mg/dL (Carpenter and Coustan [24]).

Further comparisons between women with GDM and without GDM (Supplemental Table S1) revealed that most women with GDM had a family history of diabetes (*p* < 0.001) and gained weight above IOM recommendations for her BMI (*p* < 0.048) Fig. 1 describes the fasting blood glucose in T1 and T3. The percentage of women who had FBG ≥ 100 mg/dL was more prevalent in T3 (23%) compared to T1 (17%), while the percentage of FBG ≥ 126 mg/dL was higher in T3 (3.7%) compared to T1 (1.8%). None of the maternal characteristics differed between women having normal FBG < 100 mg/dL vs. women having FBG ≥ 100 mg/dL (Supplemental Table S2). Daily consumption of vegetables was higher in the normal FBG group compared to FBG ≥ 100 mg/dL (*p* < 0.0019), while dried fruits consumption was higher in the FBG ≥ 100 mg/dL vs. FBG < 100 mg/dL (*p* < 0.0413) in T1 only (Supplemental Table S3).

### Maternal characteristics per trimester

Table 2 presents GWG, lifestyle and psychosocial factors in each trimester. Smoking incidence was less than 9% and did not differ across trimesters. Although most women were sedentary across trimesters, there was a significant decline in PA between the first and third trimesters (*p* < 0.001). Among the psychosocial variables, stress and sleep quality significantly differed across trimesters. The greatest percentage of women with high stress was reported in T3 (20.52%) (*p* < 0.001). The PSQI also showed the greatest percentage of poor sleep in T3 compared to T1 and T2 (*p* < 0.001). With respect to adherence to the LMeD, a higher percentage of women (26.0%) had low adherence scores in T3 compared to T1 and T2 (19.0% and 24.0% respectively) (*p* < 0.001), and in T2 more women were classified in the high adherence group (30.0%) compared to T1 (21.0%) and trimester 3 (25.0%). Among the food groups, the highest consumption of dairy products was reported in T2 (*p* < 0.0350), followed by vegetables in T2 (*p* < 0.0440).

**Table 2:** GWG, Lifestyle and Psychosocial Factors in Trimester 1, 2 and 3 in a National Sample of Pregnant Women in Lebanon.

Variables	Trimester 1	Trimester 2	Trimester 3	
	Mean ± SD; %	Mean ± SD; %	Mean ± SD; %	p
^*^ GWG				
Low	48.95	29.48	35.70	0.256
Adequate	23.24	25.0	22.0	
Excessive	27.70	45.52	42.30	
**Lifestyle Characteristics**				
**Maternal Dietary Intake**				
Mean Adherence Score	17.47 ± 3.4	17.43 ± 4.04	17.5 ± 4.46	
Categories of Adherence				
Low (Score 9-15)	12.7 ± 1.3	11.8 ± 1.9	11.5 ± 2.0	< **0.001**†
	19.0	24.0	26.0	
Medium (Score 16-20)	17.3 ± 1.7	17.7 ± 1.7	17.7 ± 1.6	
	60.0	51.0	43.0	
High (Score 21-27)	22.4 ± 1.4	22.4 ± 1.3	22.6 ± 1.5	
	21.0	25.0	30.0	
^‡^ **Food Group Intake per Day, Serving Size**				
Burghol, 1cup	0.12 ± 0.23	0.13 ± 0.22	0.13 ± 0.22	0.43
Starchy Vegetables, 1 cup	0.40 ± 0.48	0.36 ± 0.37	0.32 ± 0.36	0.256
Vegetables, 1 cup	1.79 ± 1.17	1.90 ± 1.43	1.64 ± 1.22	0.044
Fruits, 1 piece	2.44 ± 1.83	2.36 ± 1.76	2.13 ± 1.52	0.560
2 Dried Fruits, 1 serv	0.18 ± 0.45	0.18 ± 0.45	0.19 ± 0.45	0.875
2 Dairy products, 1 serv	2.36 ± 1.58	1.56 ± 1.28	1.51 ± 1.33	0.035
Olive oil, 1 tsp	1.05 ± .84	1.08 ± 0.87	1.32 ± 1.03	0.567
Eggs, 1 large	0.33 ± 0.44	0.36 ± 0.49	0.36 ± 0.48	0.789
Legumes, 1 cup	0.25 ± 0.32	0.28 ± 0.35	0.26 ± 0.30	0.567
**Smoking**				
Yes	8.99	7.29	5.85	0.212
No				
^§^**Physical Activity**				
Sedentary	84.53	89.48	94.81	<**0.001†**
Active	15.47	10.52	5.19	<**0.001†**
**Psychological Factors**				
**Perceived Stress Score**				
Low stress (Score ≤13)	5.0 ± 2.8	6.5 ± 2.7	7.3 ± 2.8	<**0.001†**
	14.24	13.57	13.45	
Moderate Stress (Score 13-26)	16.2 ± 3.4	16.38 ± 2.5	16.2 ± 1.6	
	74.76	68.73	63.28	
High stress (Score ≥27)	29.0 ± 0.0	29.5 ± 1.1	30.5 ± 2.5	
	11.00	17.7	23.28	
**Edinburgh Depression Score**				
Depressed (Score≥10)	14.0 ± 2.5	13.5 ± 3.5	14.1 ± 3.3	0.897
	42.10	39.06	37.99	
Non-depressed (Score<10)	6.0 ± 2.5	6.5 ± 2.8	6.0 ± 2.6	
	57.90	60.94	62.01	
**Pittsburgh Sleep Quality Index**				
Bad sleep (score ≥5)	7.7 ± 3.2	8.2 ± 3.1	8.64 ± 3.0	<**0.001**
	49.24	66.72	73.56	
Good sleep (Score<5)	2.8 ± 1.1	3.2 ± 0.8	3.4 ± 0.8	
	50.76	33.28	26.44	

Sample size=618 Values are means ± standard deviation (SD) if normally distributed, median (min-max) if not normally distributed, or percentages (%) if binary, unless otherwise specified.

*For 1st trimester: above 2 kgs or below 1 kg was considered excessive or low, respectively for all categories of BMI. For 2nd and 3rd trimester, the recommended weight gain per week for each BMI category is the following: 0.45–0.6 kg/wk for underweight women, 0.35–0.45 kg/wk for normal weight women, 0.2–0.3 kg/wk for both overweight, and obese women. Weight gain below or above these values was classified as low or excessive, respectively (Rasmussen et al., 2009).

^†^ Bold values (*p* < 0.001) indicate significant associations using McNemar test for proportions.

^‡^Food group intake was reported as the daily average number of servings consumed for each food group of the MeD.in each trimester: 2 1 ex for dried fruits (2 tbsp raisins or cranberries, 2 pieces dates, 4 pieces apricots), and dairy products (1 cup milk or yogurt, 1 slice cheese or 2 tbsp labneh).

^§^ Sedentary was defined as engaging in physical activity for less than 1-2 times/week for <20 min duration, active was defined as having greater than 1-2 times/week for >20 min duration.

*IGT* impaired glucose tolerance, *LMeD* Lebanese Mediterranean diet, *tsp* teaspoon.

### Determinants of gestational diabetes

Table 3 presents the multiple logistic regression models describing the predictors of GDM. In *Model 1-Maternal Characteristics*, the following variables were associated with increasing the odds of having GDM: T2 GWG [OR = 1.057, *p* < 0.0246], family history of diabetes [OR = 3.79, *p* < 0.0012] and MAP [OR = 1.057, *p* < 0.0311]. In *Model 2-Adherence to the LMeD*, the significant variables in model 1 (T2 GWG, family history of diabetes and MAP) were included in addition to the adherence to the LMeD. Results from model 2 showed that only family history of diabetes [OR = 3.672, *p* < 0.0010] and MAP [OR = 1.066, *p* < 0.0093] were positively associated with GDM, with no effect of adherence to the LMeD. In *Model 3-Identification of Food Groups*, significant variables from model 2 (family history of diabetes and MAP) were entered, in addition to adherence to the LMeD and each food group in an individual model. No food groups had an association with GDM (*p* > 0.05).

**Table 3:** Multiple Logistic Regression Models (MLR) Describing Predictors of GDM in a National Sample of Pregnant Women in Lebanon in Trimester 2 (*N* = 569).

^*^ Model 1-Maternal Characteristics †			
GDM	OR	95% CI	*p*
Trimester 2 GWG, Kg	1.057	1.014-1.226	**0.024**^‡^
Pre-pregnancy BMI, Kg/m^2^	1.078	0.996-1.167	0.062
Family History of Diabetes			
Yes	3.79	1.69-8.48	**0.001** ^‡^
No			
Pittsburgh Sleep Quality Index, Trimester 2			
Poor Sleep (Score<5)	0.592	0.258-1.360	0.216
Good Sleep (Score>=5)			
Edinburgh Depression Scale, Trimester 2			
Depressed (Score≥10)	1.215	0.552-2.677	0.628
Non-Depressed (Score<10)			
Perceived Stress Score, Trimester 2			
High (Score ≥27)	0.634	0.160-2.516	0.516
Medium (Score 13-26)	0.601	0.201-1.792	0.361
Low (Score ≤13)			
MAP Trimester 2, mmHg	1.057	1.005-1.112	**0.031** ^‡^
^*^ **Model 2-Adherence to the LMeD †**			
Adherence to the LMeD, Trimester 2			
High (Score 21-26)	0.676	0.213-2.438	0.507
Medium (Score 16-20)	0.894	0.343-2.331	0.818
Low (Score 9-15)			
Family History of Diabetes			
Yes	3.673	1.694-7.964	**0.001** ^‡^
No			
MAP Trimester 2, mmHg	1.066	1.016-1.119	**0.009** ^‡^
^*^ **Model 3-Identification of Food Groups†**			
Adherence to the LMeD, Trimester 2			
High (Score 21-26)	0.676	0.213-2.438	0.507
Medium (Score 16-20)	0.894	0.343-2.331	0.818
Low (Score 9-15)			
Family History of Diabetes			
Yes	3.673	1.694-7.964	**0.001** ^‡^
MAP Trimester 2, mmHg	1.066	1.016-1.119	**0.009** ^‡^

*Sample size: n = 560 in model 1, and n = 569 in model 2 and model 3.

† Model 1 represents the logistic model of the variables: family history of diabetes, pre-pregnancy BMI, GWG, stress, sleep, depression and MAP with the outcome GDM in trimester 2. Model 2 represents the significant variables in model 1 (T2 GWG, family history of diabetes, MAP) and adherence to the LMeD. Model 3 represents the food groups that were significant.

^‡^ Bold values (*p* < 0.05) indicate significant associations.

### Determinants of impaired glucose tolerance

Table 4 presents the multiple logistic regression models and predictors of impaired FBG ≥ 100 mg/dL in T1 and T3. For T1, perceived stress entered in *Model 2* and *Model 3* and was associated with impaired FBG ≥ 100 mg/dL [OR = 2.885, *p* < 0.0392; OR = 3.054, *p* < 0.0296] respectively. Adherence to the LMeD, pre-pregnancy BMI, sleep, depression and MAP did not enter in any model. However, in *Model 3 for T1*, two food group models emerged; both burghol [OR = 3.023, *p* < 0.0361] and legumes [OR = 2.100, *p* < 0.0361] were associated with an incre ased likelihood of an OR for FBG ≥ 100 mg/dL.

**Table 4:** Multiple Logistic Regression (MLR) Describing Predictors of Impaired FBG ≥ 100 mg/dl in a National Sample of Pregnant Women in Lebanon in Trimester 1 (*N* = 543) and Trimester 3 (*N* = 525).

	^*^ Trimester 1 ^†^			^‡^ Trimester 3 ^§^		
Model 1-Maternal Characteristics						
FBG ≥ 100 mg/dl	OR	95% CI	*p*	OR	95% CI	*p*
GWG, kg	0.972	0.909-1.039	0.401	1.036	1.001-1.072	**0.044**^||^
Pre-pregnancy BMI, kg/m^2^	0.977	0.919-1.039	0.459	0.990	0.944-1.037	0.667
Family History of Diabetes, Yes	1.376	0.741-2.551	0.312	2.842	0.875-2.232	0.161
Pittsburgh Sleep Quality Index						
Poor Sleep (Score<5)	1.118	0.645-1.937	0.692	1.136	0.668-1.931	0.638
Good Sleep (Score > =5)						
Edinburgh Depression Scale						
Depressed (Score≥10)	0.656	0.382-1.126	0.126	1.176	0.783-1.766	0.434
Non-Depressed (Score<10)						
Perceived Stress Score						
High (Score ≥27)	2.842	0.987-8.193	**0.053**^||^	1.152	0.565-2.349	0.697
Medium (Score 13-26)	1.385	0.590-3.252	0.454	1.404	0.752-2.622	0.286
Low (Score ≤13)						
MAP, mmHg	1.019	0.986-1.054	0.259	1.014	0.990-1.039	0.246
**Model 2-Adherence to the LMeD**						
Adherence to the LMeD						
High (Score 21-26)	0.978	0.407-2.349	0.959	0.905	0.510-1.605	0.733
Medium (Score 16-20)	1.339	0.656-2.735	0.422	1.249	0.735-2.122	0.410
Low (Score 9-15)						
GWG, Kg	--	--	--	1.036	1.003-1.070	**0.033**^||^
Perceived Stress Score						
High (Score 21-26)	2.885	1.053-7.743	**0.039**^||^	--	--	--
Medium (Score 16-20)	1.355	0.588-3.125	0.476 --	--	--	
Low (Score ≤13)						
**Model 3-Identification of Specific Food Groups**						
Adherence to the MeD	--	--	--	--	--	--
Perceived Stress Score						
High (Score≥27)	2.605	0.948-7.155	0.063	--	--	--
Medium (Score 13-26)	1.369	0.592-3.162	0.462	--	--	--
Low (9-13)						
**Legumes, serving/day**	2.100	1.049-4.205	**0.036**^||^	--	--	--
GWG, Kg	--	--	--	1.036	1.003-1.071	**0.032**^||^
Perceived Stress Score						
High (Score ≥27)	3.054	1.117-8.354	**0.029**^||^	--	--	--
Medium (Score 13-26)	1.390	0.600-3.221	0.443	--	--	--
Low (Score≤13)						
**Burghol, serving/d**	3.023	1.190-7.679	**0.020**^||^	--	--	--
Adherence to the MeD	-	-	NS	-	-	NS
Perceived Stress Score	--	--	--	-	-	NS
**Vegetables, serving/d**	--	--	--	0.742	0.587-0.936	**0.012**

^*^ Trimester 1 Sample size= 543.

^†^ Model 1 represents the logistic model of the variables: family history of diabetes, pre-pregnancy BMI, GWG T1, stress T1, sleep T1, depression T1 and MAP T1 with the outcome impaired FBG (≥100 mm Hg).

^†^ Model 2 represents the significant variables in model 1 (stress) and adherence to the LMeD in T1.

^†^ Model 3 represents the significant variables in model 2 (stress T1), adherence to the LMeD T1 and significant food groups (burghol and legumes) T1.

^‡^ Trimester 3 Sample size: n = 525 in model 1, and n = 528 in model 2 and 3.

^§^ Model 1 represents the logistic model of the variables: family history of diabetes, pre-pregnancy BMI, total GWG, stress T3, sleep T3, depression T3 and MAP T3 with the outcome impaired FBG in T3.

^§^ Model 2 represents the significant variable total GWG in model 1 and adherence to the LMeD.

^§^ Model 3 represents the significant variables in model 2: total GWG, adherence to the LMeD and significant food group (vegetable) in T. The results shown represent the values for the significant variables only.

|| Bold values (*p* < 0.05) indicate significant associations with FBG ≥ 100.

- Indicates non-significant associations with FBG ≥ 100.

--Variable not included in the model.

*OR* odds ratio for the logistic model, *95% CI* 95% Wald Confidence Limits, *NS* non-significant.

During T3, total GWG was associated with increased impaired FBG ≥ 100 mg/dL in *Model 1* [OR = 1.036, *p* < 0.0444] *and Model 2* [OR = 1.036, *p* < 0.0334]. In *Model 3*, total GWG remained significant [OR = 1.036, *p* < 0.0321], and vegetables emerged where higher intakes decreased IGT risk [OR = 0.742, *p* < 0.0120]. Adherence to the LMeD was not associated with IGT.

## Discussion

This study was the first national longitudinal study in the MENA region to investigate predictors of GDM and IGT among pregnant Lebanese women and uncovered several key factors influencing their risk. GDM incidence in this population was found to be 5.6%, which is lower than what is reported in other MENA countries (14%). On the other hand, IGT prevalence was higher compared to other MENA countries with 17% in T1 and rose to 24% in T3 [12].

The discrepancy in GDM prevalence between our study and previous studies from the MENA region may reflect divergence in the overall burden of adult diabetes versus pregnancy-specific outcomes. Moreover, the combination of low GDM incidence and high IGT may stem from differences in screening protocols across countries, or the presence of subclinical dysglycemia in our population, where fewer cases meet the diagnostic thresholds for GDM as defined by the oral glucose tolerance test (OGTT).

We have used a culturally adapted and validated dietary tool for our population that is representative of their dietary intake to assess for adherence to a Middle Eastern version of the MeD, or LMeD [13]. Burghol, eggs and dried fruits are identified as main components of the LMeD only and are not commonly found in other MeD indices [13]. Adherence to the LMeD was not associated with either outcome, at any point during pregnancy, while the majority of previous studies showed a significant association between MeD and GDM [3, 5], including in low-risk populations [4]. This could be explained by the good compliance to the LMeD by most participants in the present study, with only 20% or less showing low adherence. The latter finding, together with the lower prevalence of GDM found in the study, suggest that the diet may offer protective benefits against GDM, although other factors present in our sample such as family history, eMAP, stress, and excessive GWG were more significant predictors. Also, it is possible that diet alone was insufficient to counteract the combined effects of these factors on impaired FBG.

Sleep quality and depression did not emerge as significant predictors of GDM and IGT in the present study, unlike what has been reported previously [8]. This could be explained by the high percentage of women (60%) without depression found in the present study throughout the trimesters. On the other hand, family history of diabetes was significantly associated with GDM in trimester 2 and increased the odds of having GDM by 3.7 times. Consistently, one meta-analysis [27] reported that women with a family history of diabetes were twice as likely to develop GDM. Excessive GWG in trimesters 2 and 3, was also significantly associated with GDM and FBG, in line with two recent reviews [28, 29]. Although Mediterranean women are often reported to start pregnancy with a BMI ≥ 25 kg/m2 and experience excessive GWG, which contributes to higher IGT and GDM rates [12, 30], the present study did not find a direct relationship between pre-pregnancy BMI, GDM and IGT. This could be explained by the fact that more than 63% of the women had a normal pre-pregnancy BMI, whereas excessive GWG was more prevalent (42 and 45% in T2 and T3, respectively). This further suggests that adherence to a MeD might be associated with a lower pre-pregnancy BMI [31].

Although previous studies have consistently highlighted the role of SBP and DBP on GDM risk [32], our study was among the first few studies to show significant associations between eMAP and GDM risk, in line with the literature [33]. Hypertensive disorders are more common among women with GDM and can occur in subsequent pregnancies prior to GDM diagnosis, as reported in a study by Lee et al. [27]. This can be explained by the shared pathophysiology of both conditions, where elevated insulin resistance and impaired endothelial function contribute to dysregulated glucose metabolism and vascular dysfunction, thereby increasing the risk of both GDM and hypertensive disorders of pregnancy (HDPs) [34]. Moreover, in a recent meta-analysis on the effect of different diets on GDM including the MeD diet and the Dietary Approaches to Stop Hypertension (DASH), the latter showed superiority to the MeD [27] and aligns with our observation about eMAP as a determinant of GDM.

While adherence to LMeD did not predict GDM or IGT, intake of specific food groups showed significant associations, where a decrease in the intakes of burghol and legumes and an increase in vegetable intake were protective against the development of IGT in the first and third trimesters, respectively. Burghol and legumes are high sources of carbohydrates, and their excessive intake may lead to impaired glycemia especially during pregnancy [35]. It is noteworthy to mention that food-specific associations such as burghol and legumes may be related to portion sizes, preparation methods, and total carbohydrate load rather than intrinsic harms of those food groups. Although it is recommended that women with IGT consume complex carbohydrates, the total amount of carbohydrate should not exceed 40-45% of their total caloric intake [36]. This is particularly important in the context of excessive energy intake in line with the present study’s findings, where high rates of GWG suggest overconsumption of calories, even though it was not directly assessed. On the other hand, vegetable intake was associated with lower risk of IGT in trimester 3. Vegetables are low in carbohydrate and saturated fats, and rich in antioxidants and fibers, which can reduce inflammation and improve glycemia [37].

Maternal stress was also linked to a greater IGT risk in the 1st trimester only. Similarly, one study by Mishra et al. [9] showed a 13-fold increase in GDM risk among those with high perceived stress before 24–28 weeks while another study showed a 2.5 increase in GDM risk among women who experienced stressful events in the past 12 months before delivery [38]. This can be related to stress-induced activation of the hypothalamic-pituitary-adrenal (HPA) axis leading to the release of hormones that inhibit insulin secretion and raise blood glucose levels [39]. It is also possible that the effect of poor sleep is mediated through increased stress levels in the 1st trimester, as shown in a study by Dorheim et al. [40].

Our findings underscore the importance of early interventions for pregnant women with a family history of diabetes, elevated MAP, and high stress levels. Specifically, it is crucial to limit excessive GWG and reduce the intake of high-carbohydrate foods, such as burghul and legumes, particularly during the first trimester. Furthermore, increased vegetable consumption in the third trimester is recommended. Clinicians should prioritize early screening for GDM risk factors, in particular elevated MAP, monitor GWG, and implement early dietary strategies to prevent IGT and improve pregnancy outcomes.

### Strengths and limitations

The primary strength of this study is its national scope and longitudinal design, with repeated trimester-specific measures of diet, psychosocial, and vascular factors. The use of a culturally adapted, validated dietary tool is an additional notable strength. However, several limitations should be acknowledged. First, the relatively low prevalence of GDM reduced statistical power, potentially leading to the underestimation of true effects. Second, dietary and psychosocial data were self-reported and are thus subject to recall bias, social desirability bias, and inaccuracies in reporting. Third, the study coincided with the Lebanese economic crisis and the COVID-19 pandemic (2019–2021), which may have influenced participants’ behaviors and study outcomes. COVID-19 lockdowns, movement restrictions, and social isolation likely affected physical activity, increased sedentary behavior, altered eating patterns (e.g., higher consumption of processed or comfort foods), and might have contributed to elevated stress and anxiety levels. These factors could have impacted GWG, dietary adherence, and psychosocial measures, potentially biasing associations with GDM and IGT. Finally, the focus on the LMeD excluded broader dietary factors such as caloric intake, consumption of processed foods, and nutrient-specific patterns.

## Conclusion

This national prospective cohort study identified key predictors of gestational diabetes and impaired glucose tolerance among Lebanese women, including family history of diabetes, elevated mean arterial pressure, excessive gestational weight gain, high stress, and intake of specific foods. While adherence to the Lebanese Mediterranean Diet was not directly associated with the study outcomes, increased vegetable intake was protective against Impaired Glucose Tolerance and Gestational Diabetes. Early identification of at-risk women, monitoring of weight gain, stress management, and targeted dietary guidance are essential. Future studies should incorporate broader dietary patterns and objective measures of diet, physical activity, and psychosocial factors to better inform preventive strategies.

## Supplementary information

Supplemental Tables

## Data Availability

The data that support the findings of this study are available from the corresponding author upon reasonable request.
