# Supplementary material for: Associations of the Mediterranean diet during pregnancy with impaired glucose tolerance and gestational diabetes: A national prospective cohort study in Lebanon
Source: Eur J Clin Nutr. 2026 Mar 3;80(4):397–406. doi: 10.1038/s41430-026-01708-3 (PMC13083240; doi:10.1038/s41430-026-01708-3)
Supplement: Supplementary file 1 — Supplemental Tables [file 41430_2026_1708_MOESM1_ESM.pdf]

Supplemental Table 1: Maternal Characteristics of Women having GDM vs those without GDM

Maternal Characteristics	GDM (N=32)<br>N (%) or Mean ± SD	No GDM (N=541)<br>N (%) or Mean ± SD	p
**Mean age in years** (N=618)	N= 32<br>29.5 ± 5.8	N=541<br>29.3 ± 4.9	
**Place of Residence**			
Beirut + Mount Lebanon	21 (66)	230 (43)	**0.028***
North Lebanon + Akkar	1 (3)	66 (12)	
South Lebanon + Bekaa	10 (31)	245 (45)	
**Education**			
<High school (High school or brevet)	8 (25)	116 (21)	0.634
University degree or higher	24 (75)	425 (79)	
**Occupation**			
Non-employed	16 (50)	263 (49)	0.878
Employed	16 (50)	278 (51	
**Family History of Diabetes**			
Yes	14 (44)	105 (20)	**0.001***
No	18 (56)	433 (80)	
†**Pre-pregnancy BMI**			
Normal (BMI 18.5-24.9)	8 (25)	121 (29)	0.414
Overweight (BMI 25-29.9) + Obese	24 (75)	400 (71)	
‡**Total GWG**			
Low	5 (17)	200 (38)	**0.048***
Adequate	16 (55)	234 (44)	
Excessive	8 (28)	92 (17)	

* Indicates significant associations using Chi Square test. † Pre-pregnancy BMI was calculated as weight in Kg over height in meters squared, and was categorized into underweight, normal, overweight and obese according to IOM. Among women with GDM, no one was underweight. ‡ Total gestational weight gain was calculated by subtracting total weight gained from pre-pregnancy BMI. GWG was classified as inadequate, appropriate or excessive applying the following recommendations: To be considered adequate GWG, women who are underweight should gain a total of 12.5-18 kg, normal weight between 11.5-16 kg, overweight between 7-11.5 kg, and obese between 5-9 kg. Those with low GWG have a weight gained below these recommendations whereas those with excessive weight gain have above these recommendations (American College of Obstetricians and Gynecologists, 2013).

Supplemental table 2. Comparison of Maternal Characteristics among Women in the Normal (FBG<100 mg/dl) vs. Impaired Glucose Tolerance (IGT) (FBG≥100 mg/dl) in Trimesters 1 and 3 in a National Sample of Pregnant Women in Lebanon (N=618)

	**Trimester 1**			**Trimester 3**		
	**Control**	**IGT**		**Control**	**IGT**	
	**Mean (SD); %**	**Mean (SD); %**	**p**	**Mean (SD); %**	**Mean (SD); %**	**p**
**Health Status**						
Mean FBG Values (mg/dl)	90.3±4.9	109.6 ±11.6	0.214	90.4 ±4.2	109.5±11.2	0.345
	88.0	12.0		73.0	27.0	
Pre-pregnancy BMI, kg/m^2						
<18.5	17.1±1.1	18.0±0.4	0.562	17.1±14.7	18.0±0.4	0.759
	4.0	2.0		4.0	0.0	
18.5-24.9	21.9±1.8	22.0±1.6		21.9±1.8	21.9±1.6	
	62.0	65.0		62.0	57.0	
≥30	29.2±3.7	30.3±4.1		29.3±3.7	29.2±3.9	
	34.0	33.0		34.0	43.0	
Mean GWG, Kg	1.9±4.4	1.5±4.3		14.0±5.8	15.2±6.2	
	88.0	12.0		73.0	27.0	
* GWG Categories						
Low	1.4±3.2	0.86±3.0	0.288	8.6±3.3	9.1±3.7	0.234
	97.0	96.0		37.0	33.0	
Adequate	15.2±1.4	16.0±1.7		15.3±1.7	15.5±1.7	
	2.0	4.0		46.0	44.0	
Excessive	25.1±2.6	27.1 ± 3.4		22.9±4.7	23.6±4.7	
	1.0	0.0		16.0	23.0	
†Mean MAP, mmHg	82.9±7.7	84.0±8.0		86.0±8.1	86.8±7.8	
	88.0	12.0		74.0	26.0	
MAP						
Not High	79.6±5.0	80.2±5.3	0.345	80.2±3.6	80.2±3.7	0.103
	78.0	73.0		64.0	46.0	
High	94.2±4.0	94.2±4.0		92.8±6.6	92.5±5.6	
	22.0	27.0		46.0	54.0	
**Psychological Factors**						
Perceived Stress Score						
Low (Score≤13)	9.6±2.8	9.9±3.8	0.061	10.2±2.5	9.7±2.6	0.191
	15.0	10.0		14.0	13.0	
Moderate (Score 14-26)	20.3±3.5	20.6±3.5		20.3±3.4	20.7±3.5	
	75.0	70.0		61.0	65.0	
High (Score≥27)	29.4±2.3	29.6±2.2		30.9±2.9	30.0±2.3	
	10.0	19.0		24.0	22.0	
Pittsburgh Sleep Quality						
Good Sleep (Score<5)	2.8±1.2	7.0±3.1	0.440	3.3±0.8	3.5±0.8	0.615
	48.0	43.0		17.0	16.0	
Poor Sleep (Score≥5)	7.7±3.1	7.0±3.1		8.5±3.0	8.8±3.2	

	52.0	57.0		83.0	84.0	
Edinsburgh Depression Score						
Depressed (Score≥10)	14.0±3.5	14.8±4.8	0.229	14.2±3.4	14.6±3.8	0.575
	47.0	39.0		43.0	46.0	
Non-depressed (Score<10)	5.9±2.6	6.2±2.1		6.0±2.7	5.4±2.7	
	53.0	61.0		57.0	54.0	

Sample size=618. Values are means ± standard deviation (SD) if normally distributed, median (min-max) if not normally distributed, or percentages (%) if binary, unless otherwise specified.

[*] Total gestational weight gain was calculated by subtracting total weight gained from pre-pregnancy BMI. GWG was classified as low, adequate or excessive applying the following recommendations: To be considered adequate GWG, women who are underweight should gain a total of 12.5-18 kg, normal weight between 11.5-16 kg, overweight between 7-11.5 kg, and obese between 5-9 kg. Those with low GWG have a weight gained below these recommendations whereas those with excessive weight gain have above these recommendations (American College of Obstetricians and Gynecologists, 2013).

[†] Not high MAP in trimester 1 is ≤ 87 mm Hg, and high MAP > 87 mm Hg, and in trimester 3 not high MAP is ≤ 86 mm Hg,and high MAP is >86 mm Hg (Women's health and education center, 2009).*Indicates significant associations using McNemar test for proportions.

Abbreviations: IGT, impaired glucose tolerance, MeD, IGT, impaired glucose tolerance, FBG, fasting blood glucose, GWG, gestational weight gain, MAP mean arterial pressure

Supplemental table 3. Comparison of Dietary Intake among Women in the Normal (FBG<100 mg/dl) vs. IGT Group (FBG≥100 mg/dl) in Trimesters 1 and 3 in a National Sample of Pregnant Women in Lebanon (N=618)

**Dietary Variable**	**Trimester 1**			**Trimester 3**		
	**Control**	**IGT**		**Control**	**IGT**	
	**Mean (SD); %**	**Mean (SD); %**	**p**	**Mean (SD); %**	**Mean (SD); %**	**p**
**Adherence to the MeD**						
Low (Score 9-15)	12.8±1.2	13.3±1.0	0.806	12.4±1.8	12.0±1.4	0.406
	17.6	20.9		22.4	23.8	
Medium (Score 16-20)	17.2±1.7	17.7±1.7		17.7±1.6	17.7±1.6	
	60.6	55.8		45.0	57.1	
High (Score 21-27)	22.3±1.3	22.7±2.2		22.6±1.5	22.4±1.4	
	21.8	23.2		32.5	19.0	
***Food Group Intake per Day, Serving Size**						
Burghol, 1 cup	0.11+-0.19	0.22+-0.48	0.212	0.12+-0.17	0.16+-0.32	0.135
Starchy Vegetable, 1 cup	0.54+-0.97	0.39+-0.40	0.433	1.59+-1.02	1.81+-1.23	0.278
Vegetable, 1 cup	1.95+-1.29	1.79+-1.16	**0.032**†	1.58+-1.02	1.81+-1.23	0.795
Fruit, 1 item	2.75+-2.35	2.47+-1.80	0.672	2.28+-1.57	2.26+-1.46	0.875
Dried Fruits, 1 serv	0.06+-0.17	0.19+-0.48	**0.001**†	0.21+-0.49	0.20+-0.46	0.721
Dairy products, 1 serv	2.53+-1.59	2.38+-1.62	0.118	1.62+-1.33	1.62+-1.32	0.965
Olive oil, 1 tsp	0.95+-0.75	1.07+0.83	0.205	1.36+-0.89	1.43+-1.08	0.458
Eggs, 1 large	0.33+-0.44	0.32+-0.42	0.632	0.39+-0.53	0.39+-0.47	0.747
Legumes, 1 cup	0.37+-0.63	0.24+-0.29	0.111	0.28+-0.28	0.26+-0.29	0.622

Sample size= 618

*Food group intake was reported as the daily average number of servings for each food group of the MeD.: 1 serving for dried fruits (2tbsp raisins or cranberries, 2 pieces dates, 4 pieces apricots), and 1 ex for dairy products (1 cup milk or yogurt, 1 slice cheese or 2 tbsp labneh).

†Indicates significant associations using McNemar test for proportions.

Abbreviations: IGT, impaired glucose tolerance, MeD, Mediterranean diet, tsp, teaspoon.
